# Mechanistic Understanding of Superior Methylene Blue Adsorption Capacity in a Novel g-C_3_N_4_ Modified Amorphous Na–Ca–Mg Silicate Adsorbent: Insights from Multinuclear Solid-State NMR Spectroscopy

**DOI:** 10.1021/acs.jpcb.4c06514

**Published:** 2024-12-09

**Authors:** Sezen Soyer-Uzun, Ping Yu, Feyza Kevser Öner, Sabyasachi Sen

**Affiliations:** † Department of Chemical Engineering, Bogazici University, Istanbul, Bebek 34342, Türkiye; ‡ Nuclear Magnetic Resonance Facility, University of California, Davis, California 95616, United States; § Department of Materials Science and Engineering, University of California, Davis, California 95616, United States

## Abstract

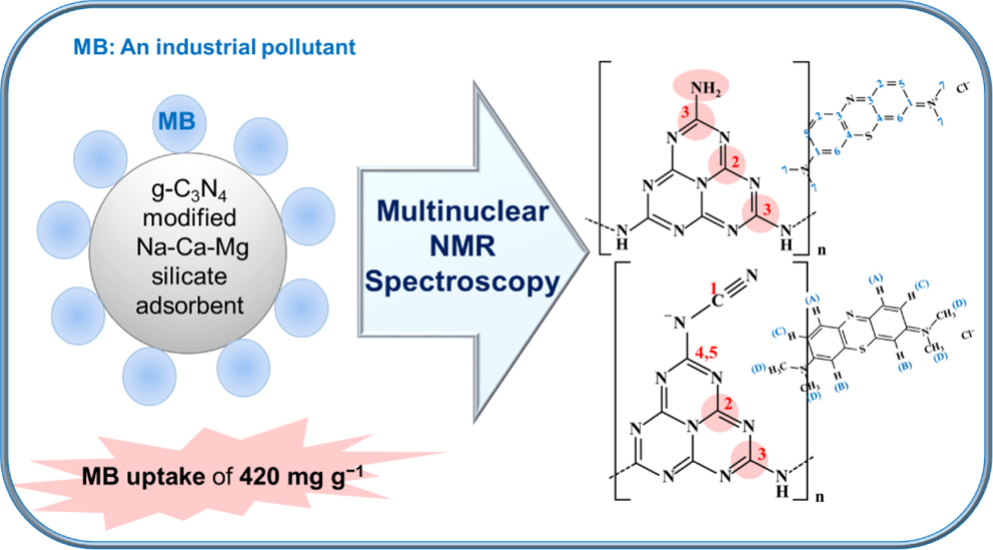

Silicate-based adsorbents offer significant advantages over traditional materials, particularly due to their superior thermal and chemical stability, enhanced regenerability, and the ability to endure more rigorous operating conditions. In this study, an amorphous Na–Ca–magnesium silicate adsorbent (SAAM) and its g-C_3_N_4_-modified counterpart (gCN-SAAM) were synthesized via alkali activation and a subsequent thermal process, respectively. The g-C_3_N_4_ modification resulted in a novel hybrid adsorbent with a remarkable methylene blue (MB) adsorption capacity of 420 mg g^–1^, four times higher than the unmodified sample, setting a new benchmark. Solid-state ^29^Si (MAS and CP/MAS), ^1^H MAS, and ^13^C CP/MAS NMR spectroscopy were used to investigate the complex structures of these adsorbents and their interactions with MB. The local structure of SAAM primarily consists of Q^3^ Si units, with minor Q^0^ and Q^1^ Si species, structural water, and Mg–OH sites. Exposure to MB caused an upfield shift in the ^29^Si CP/MAS spectrum and enhanced resonances in the high-field region, indicating MB interaction with Si sites. ^1^H MAS NMR spectra revealed significant interactions between water molecules in the geopolymer-like framework of SAAM and MB. The thermal treatment of SAAM with urea to produce gCN-SAAM enhanced the polymerization of Q^3^ Si species and increased the relative fraction of Q^4^ Si sites. This treatment also reduced the intensity of some Mg–OH units, showing interaction with g-C_3_N_4_. After MB adsorption on gCN-SAAM, NH_2_ groups of g-C_3_N_4_ disappeared, and shifts in the C_2N-NHx_ and C_3N_ sites indicated their involvement in adsorption, while Si sites remained intact. This thermal method creates a sustainable, cost-effective and efficient adsorbent for MB removal from wastewater. Multinuclear NMR spectroscopy provides detailed insights into the adsorbent’s complex structure and MB interactions, potentially guiding the design of improved future adsorbents.

## Introduction

Water pollution with synthetic dyes presents a multifaceted environmental crisis with significant global ramifications. The extensive use of synthetic dyes across industries such as textiles, paper, plastics, and cosmetics has led to widespread contamination of water bodies worldwide. Worldwide production of dyes and pigments exceeds 700,000 tons annually, encompassing approximately 10,000 distinct types.^1^ Roughly 10% of this volume ends up in wastewater. Among these dyes, methylene blue (MB) is a widely used one, particularly employed in the textile, leather and pharmaceutical sectors in dyeing fabrics and as a biological stain. Due to the substantial scale of industrial usage, a significant amount of wastewater containing MB is released into both groundwater and surface water sources. MB persists in the environment for extended periods, further aggravating its damaging effects.^2^ The environmental impact extends beyond aquatic ecosystems, as contaminated water sources can infiltrate agricultural systems, affecting soil quality and crop productivity. In the case of leaching in drinking water, MB poses serious health risks, with potential carcinogenic and mutagenic effects on human populations.^3^ When administered at excessive doses, the monoamine oxidate inhibitory properties of MB can lead to fatal serotonin toxicity in humans, besides posing a threat to aquatic fauna and the overall aquatic ecosystem.^4^

Adsorption is a widely employed, cost-effective and practical method for dye elimination from aqueous solutions. It is particularly advantageous for treating wastewater with low concentrations, where other methods may be less effective. Ideally, a good adsorbent is expected to display certain properties such as high adsorption capacity, economic feasibility, simple synthesis, easy recovery, regenerability, and chemical stability. In addition to these properties, strong mechanical performance would be extremely valuable for practical applications in scaled-up processes such as utilization in fluidized bed reactors, and in the recovery and potential usage in water storage and purification. Various adsorbents, such as activated carbon and composites,^5−8^ bioadsorbents,^9^ metal organic frameworks, membranes, agricultural or industrial solid wastes,^10−12^ and clays^13^ have been investigated toward MB elimination from solutions. However, all these systems either lack mechanical strength or they are synthesized using complex processes. At this point, alkali-activated materials, commonly known as geopolymers, have emerged as effective alternatives due to their excellent mechanical performance and sustainability benefits.

Although terms such as “alkali-activated materials”, “geopolymers”, and “inorganic polymers” are frequently used interchangeably, one should recognize that “geopolymer” refers specifically to a three-dimensional network with the most aluminum and the least calcium content among these materials.^14^ These materials offer advantages toward sustainability and circular economy due to their energy-efficient synthesis procedures and utilization of industrial wastes in geopolymerization.^15−17^ Although they have arisen as environmentally friendly alternatives to ordinary cement-based construction materials due to their excellent mechanical properties, they also have applications in coatings, fire-resistant materials, and catalyst supports^18^ as well as adsorbents and/or photocatalysts for environmental applications.^19,20^ Geopolymers and their modified or hybrid versions have been investigated extensively in the past for potential effective and economical applications in dye and heavy metal removal from aqueous solutions. For instance, red mud–metakaolin systems with the incorporation of an anatase component have been shown to demonstrate adsorption and photocatalytic activity under UV-light conditions.^21^ The adsorption capability of these red mud–metakaolin systems has been further enhanced by depositing urea-based g-C_3_N_4_ onto their surface.^22^ In another recent study, a porous steel slag-based geopolymer was shown to effectively remove Cu^2+^ ions.^23^ Another system involving KOH-activated slag-based geopolymer microspheres was reported to exhibit high adsorption capacities for recycling Co(II) compared to NaOH-activated samples.^24^ A separate investigation demonstrated the synthesis of geopolymer hybrids with polymer-conjugated zeolite phases that are capable of capturing and storing metal cations. The ^23^Na–^23^Na DQ MAS NMR, combined with DFT calculations in this study, provided an approach for atomic-scale understanding of the role of Na^+^ ions in aluminosilicate solids.^25^

Geopolymers are known as the reaction product of an aluminosilicate precursor with an alkali hydroxide and/or alkali silicate solution. During polymerization reaction dissolution of the raw materials and condensation reactions take place to lead to a dense and highly polymerized three-dimensional network composed of corner-shared AlO_4_ and SiO_4_ tetrahedral units with charge balancing Na and/or K cations.^26,27^ Although geopolymers are widely known as alkali-aluminosilicate materials, there were limited number of attempts to synthesize their magnesium analogs using the minerals talc and sepiolite.^28,29^ To the best of our knowledge, there is only one NMR spectroscopic study focusing on magnesium silicate-based geopolymer systems, in which the synthesis of magnesium-containing analogues of aluminosilicate geopolymers from the magnesiosilicate minerals chrysotile, talc, and sepiolite was investigated. The effect of pretreating these minerals by grinding and/or dehydroxylation was examined using X-ray diffraction (XRD) and ^29^Si and natural-abundance ^25^Mg solid-state magic angle spinning (MAS) NMR spectroscopy.^28^ Among the minerals studied, the products from sepiolite most closely resembled an aluminosilicate geopolymer, forming an X-ray amorphous product with a characteristic ^29^Si MAS NMR resonance at −90 ppm. The ^25^Mg MAS NMR spectrum indicated that some magnesium could be incorporated into tetrahedral sites, typical for geopolymers. Sepiolite based alkali-activated materials have recently been shown to offer great potentials as economically feasible and mechanically strong adsorbents for efficient methylene blue elimination.^29,30^ However, a detailed fundamental understanding is lacking in these systems mainly due to the disordered nature and complex atomic structures of these newly emerged adsorbents. It should be noted that an atomic scale understanding of the structure of the adsorbent, identification of the adsorption sites and their roles in the adsorption processes is a strong requirement for the design of efficient adsorbents with desired functionalities.

On the other hand, graphitic carbon nitride (g-C_3_N_4_) is a chemically stable and cost-effective material with a fairly simple synthesis procedure that has been frequently researched because of its potential applications in dye adsorption as well as in photocatalysis.^22,31^ Its high surface area provides ample active sites for adsorption, while its π-conjugated structure facilitates π–π interactions with dye molecules. Additionally, its tunable band gap allows for efficient visible light absorption offering prospective as metal-free photocatalysts for dye degradation. Although its chemical formula does not contain H or O atoms, it is likely to contain H/O containing functional groups of the precursors forming bonds with C and N atoms, resulting in a variety of functional groups during thermal polymerization of the precursors, depending on the synthesis conditions. Consequently, g-C_3_N_4_ can be designed to include a variety of functional groups such as amino (−NH_2_), nitrile (−C≡N), imine (−NH), amide (−CONH_2_), and hydroxyl (−OH) groups by employing different chemical processes and modifications.^32−35^ For instance, the presence of amino groups could provide basic properties that could interact with acidic dye molecules like MB while nitrile groups could contribute largely to the chemical stability ensuring robustness in adsorption processes.

Given the above-mentioned advantages of g-C_3_N_4_ and sepiolite-based disordered magnesium silicate systems, we are motivated to investigate their synergistic effect on MB adsorption using a hybrid adsorbent based on these two systems. Here, we introduce a novel g-C_3_N_4_-modified amorphous magnesium silicate material that exhibits an excellent adsorption capacity of 420 mg g^–1^ under ambient conditions (initial methylene concentration of C_0_ = 80 mg L^–1^). However, the primary focus of the present work is not to optimize the aforementioned performance value by tuning adsorption conditions such as pH and temperature, but rather to grasp an atomic scale understanding of the adsorbent structures and corresponding MB adsorption processes, which is lacking in many adsorption studies. The primary goals are (1) to understand the rather complex chemical structure of this g-C_3_N_4_-modified amorphous magnesium silicate adsorbent and its unmodified counterpart, in a stepwise approach from raw material to final adsorbent with a focus on the local Si, C and H chemical environments and (2) to explore the interactions of these adsorbents with MB comparatively, to build a mechanistic understanding of the adsorption process. For this purpose, we have utilized multinuclear magnetic resonance (NMR) spectroscopic techniques including ^29^Si and ^1^H magic angle spinning NMR (MAS NMR) and ^13^C–^1^H and ^29^Si–^1^H cross-polarization MAS (CP/MAS) NMR. It may be noted that in contrast to regular MAS NMR that yields quantitative information on the relative abundance of all structural sites involving the nuclide under observation, the CP/MAS NMR emphasizes signal from those sites that are in close proximity to H atoms.

## Materials and Methods

### Materials

The clay mineral sepiolite was provided by Dolsan Mining, Türkiye. It was calcined at 750 °C for 1 h before its utilization in alkali-activation.^28,29^ Chemical composition of calcined sepiolite is given in Table 1.^29^

**Table 1:** Chemical Composition (wt %) of Calcined Sepiolite^29^

**Sample**	**SiO**_**2**_	**MgO**	**CaO**	**Al**_**2**_**O**_**3**_	**TiO**_**2**_	**Fe**_**2**_**O**_**3**_	**K**_**2**_**O**	**SO**_**3**_	**SrO**
Calcined sepiolite	52.7	31.0	13.61	1.51	0.07	0.59	0.25	0.09	0.17

A mixture of sodium hydroxide (NaOH) and sodium silicate (Na_2_Si_3_O_7_) aqueous solution (9 wt % of Na_2_O, 28 wt % of SiO_2_, 63 wt % of H_2_O, Merck, 1.35 g/cm^3^) was used as the alkali-activating solution to prepare the sepiolite based geopolymer-like material (SAAM). Urea (Merck, 1.34 g/cm^3^) was employed as a precursor to obtain g-C_3_N_4_ modified SAAM, gCN-SAAM. Methylene blue hydrate (Sigma-Aldrich, purity >97%, molecular weight 319.85 g/mol) was used as the model dye (adsorbate) in measuring adsorption capacity of the prepared samples.

### Sample Preparation

Calcined sepiolite is thoroughly mixed with the alkali-activating solution to obtain a Na–Ca–magnesium silicate gel with a molar composition of Na_2_O: CaO: MgO: SiO_2_: H_2_O = 0.63:0.32:1.0:3.3:14.5 (mol:mol), excluding impurities that correspond to less than 1% of the total mixture. The gel is cured at 40 °C for 1 day and then left for 1 week at ambient conditions. Adsorption measurements are performed at the end of this curing period. The resulting sample is a hardened solid Na–Ca–Mg silicate material, referred to as SAAM in the subsequent discussion.

For g-C_3_N_4_ modification of SAAM, first, urea and crushed and sieved SAAM (200 μm) were mixed in 60:1 weight ratio and grinded for 30 min. Then, the mixture was transferred into a crucible with a lid and placed in a furnace, which was initially kept at room temperature. The temperature of the furnace was increased to 550 °C at a rate of 10 °C/min and the crucible was kept at 550 °C for 2.5 h. This process resulted in a two-phase product which could be easily separated. One of the phases was in lighter color including only C_3_N_4_ while the other phase was darker in color containing gC_3_N_4_ modified SAAM. The latter sample is referred to as gCN-SAAM in the following discussion.

### Adsorption Measurements

SAAM and gCN-SAAM adsorbents were ground into powder form and sieved (200 μm, #70 mesh sieve) before using them in MB adsorption measurements. Adsorbents (5 mg) were submerged into MB solutions (50 mL) with concentrations of 80 mg L^–1^ and stirred vigorously under dark conditions. At the end of 120 min, the concentration of the MB in these solutions were assessed using a Flame-S-UV–Vis-ES spectrometer (Ocean Optics, USA) by measuring the absorbance values at λ = 664 nm as described previously.^29,30^ The adsorption tests were carried out in triplicates, and it was seen that the adsorption capacity values could be repeated to within less than 4%. MB adsorbed materials were recovered from solutions using filtration and dried in air for 2 weeks before structural characterization studies using multinuclear NMR spectroscopy. Air drying is intentionally preferred over alternative methods, such as mild heat treatment, to preserve the adsorption state and surface chemistry and avoid thermal degradation of the dye and adsorbent material.

### X-ray Diffraction

X-ray powder diffraction (XRD) measurements were conducted using a Rigaku MiniFlex 600 X-ray diffractometer with Cu Kα radiation (λ = 1.5418 Å). The data were recorded in 2θ = 5–90° with a step size of 0.02°. The power rating of X-ray generator was set to 40 kV, 15 mA, and a D/teX ultra silicon strip detector was used.

### Thermogravimetric Analysis (TGA)

The analysis was performed using a TA Instruments TGA Q500 instrument. A 25 mg sample was placed in a platinum pan, and the temperature was gradually raised to 780 °C at a ramp rate of 10 °C/min under a nitrogen flow of 60 mL/min.

### Brunauer–Emmett–Teller (BET) Measurements

The surface area of the samples was measured using a Micromeritics ASAP 2020 Physisorption Analyzer. Prior to each measurement, approximately 400 mg of each sample was degassed under vacuum at 120 °C for 8 h. Free space measurements were conducted using helium, and volumetric N_2_ adsorption isotherms were obtained with liquid nitrogen, spanning a P/P0 range of 0.008 to 0.918, with 10 data points.

### Solid State NMR Spectroscopy

Solid state ^29^Si MAS and ^29^Si–^1^H CP/MAS, ^1^H MAS and ^13^C–^1^H CP/MAS NMR experiments were performed on SAAM and gCN-SAAM samples before and after MB adsorption. All NMR spectra were collected with a Bruker AVANCE 500 MHz NMR spectrometer equipped with an 11.7 T wide-bore magnet. For ^29^Si MAS NMR experiments the samples were loaded into 4 mm zirconia rotors and were spun at 10 kHz. The ^29^Si Larmor frequency was 99.34 MHz. A direct polarization pulse sequence with a 60 degree tip angle was used along with a recycle delay time of 60 s. Spectra were also collected with a wide range of delay times to ensure that a delay of 60 s was sufficient to obtain quantitative ^29^Si MAS NMR spectra. Approximately 256 to 1024 FIDs were averaged and Fourier-transformed to obtain spectra with a signal-to-noise ratio greater than 20. All data were processed with 30 Hz of exponential line broadening. The ^29^Si–^1^H CP/MAS NMR spectra were acquired with a two-pulse-phase-modulated (TPPM) decoupling pulse sequence, a contact time of 5 ms, and a recycle delay of 5 s. Approximately 2200 to 2300 FIDs were collected, averaged and Fourier-transformed to obtain each spectrum. The ^29^Si chemical shift in all cases was externally referenced to HY zeolite at −107.5 ppm, which was originally referenced to Tetramethyl silane at 0 ppm.

For ^1^H MAS NMR experiments the samples were loaded into 2.5 mm zirconia rotors and were spun at 26.2 kHz. A direct polarization pulse sequence was used with a 30-degree tip angle and a recycle delay of 5 s. A total of 128 FIDs were averaged and Fourier-transformed with 1 Hz of line broadening to obtain each sample spectrum. The rotor background signal was also recorded under identical conditions and was subtracted from each sample spectrum for final analysis. The ^1^H NMR was referenced to TMS at 0 ppm.

^13^C–^1^H CP/MAS NMR data were acquired with a two-pulse-phase-modulated (TPPM) decoupling pulse sequence, a contact time of 3 ms, and a recycle delay of 5 s. The Larmor frequency of ^13^C was 125.74 MHz, and the MAS spinning rate was 15 kHz. Approximately 11200 to 16384 FIDs were averaged and Fourier-transformed with 10 Hz of line broadening to obtain each sample spectrum. The ^13^C chemical shift was referenced to that of the carboxyl group of glycine at 176 ppm.

## Results and Discussion

**Figure 1:**
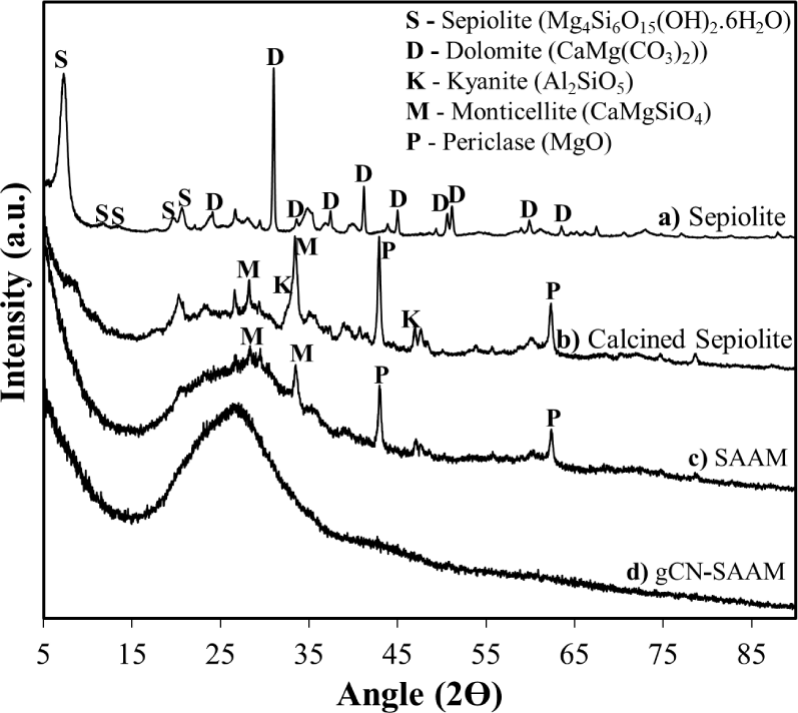
XRD patterns of a) Sepiolite in the as-received form, b) Calcined sepiolite, c) SAAM, and d) gCN-SAAM.

The XRD patterns of the as-received sepiolite, calcined sepiolite, SAAM, and gCN-SAAM are shown in Figure 1. The XRD pattern of the as-received sepiolite (Figure 1a) displays features corresponding to the sepiolite mineral, along with a dolomite impurity. Calcination at 750 °C leads to the disappearance of sepiolite peaks due to dehydration and dehydroxylation, the decomposition of dolomite, and significant amorphization, as evidenced by the broad feature between 2θ values of 20° and 40° (Figure 1b), consistent with previous observations.^28,29^ The crystal phases coexisting with the disordered structure of the calcined material include kyanite, monticellite, and periclase (Figure 1b). The reaction product of calcined sepiolite with the alkali-activating solution is SAAM; its XRD pattern (Figure 1c) exhibits a predominantly amorphous character, characterized by a featureless hump at around 28°, in agreement with previous studies. Urea treatment of SAAM at 550 °C results in a completely X-ray amorphous material, gCN-SAAM, displaying a distinct broad feature at around 27° (Figure 1d).

**Figure 2:**
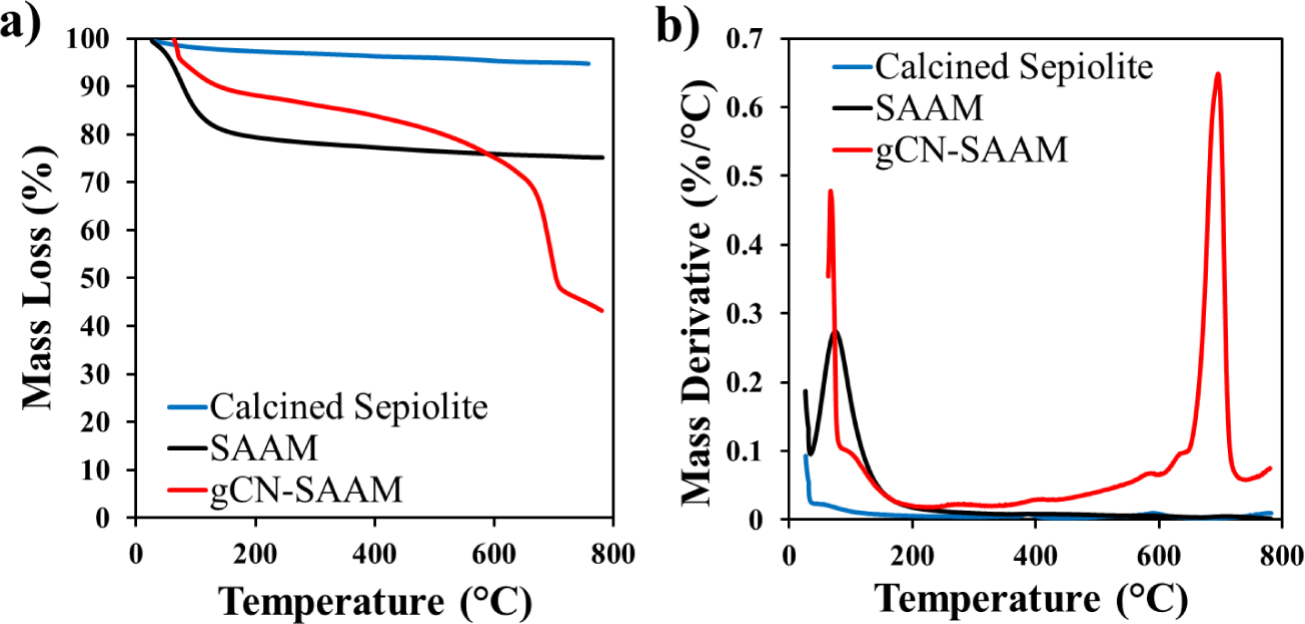
a) TG and b) DTG curves of calcined sepiolite, SAAM, and gCN-SAAM.

TG and DTG curves of calcined sepiolite, SAAM, and g-CN-SAAM are shown in Figure 2. Mass losses taking place during a temperature ramp to 780 °C were determined as 5.3, 24.9, and 56.8 wt % for calcined sepiolite, SAAM, and gCN-SAAM, respectively (Figure 2a). SAAM loses most of its water content, about 20 wt %, below 200 °C as a result of loss of adsorbed water (Figure 2b). On the other hand, the mass loss for gCN-SAAM, is observed in two main regions, below 200 °C and between 550 and 760 °C. gCN-SAAM loses 12% of its mass below 200 °C; which is thought to be related with adsorbed water. The main mass loss for gCN-SAAM, corresponding to about 35 wt %, takes place in the temperature range of 550–760 °C, which is related to the decomposition of g-C_3_N_4_.^36^ Therefore, it is estimated that gCN-SAAM contains around 35 wt % of graphitic carbon nitride component.

The BET surface area values for SAAM and gCN-SAAM are 20.1 m^2^/g and 28.2 m^2^/g, respectively. These values are much lower compared to the surface areas of their precursors, sepiolite and calcined sepiolite, which have previously reported values of 251.8 m^2^/g and 101.8 m^2^/g, respectively.^29^

### Structural Characterization of Starting Materials: Sepiolite and Calcined Sepiolite

**Figure 3:**
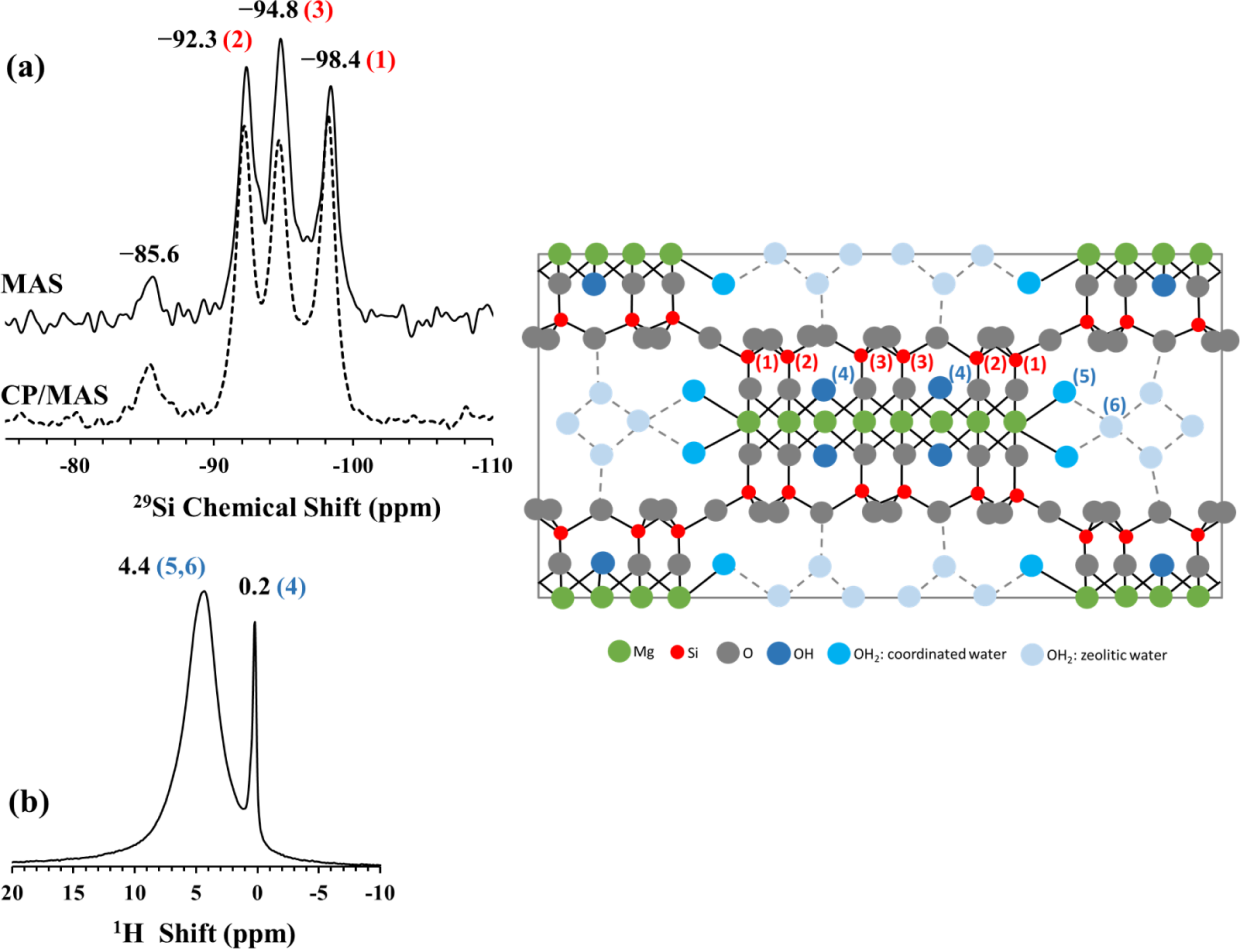
(a) ^29^Si MAS (solid line) and CP/MAS (dashed line) NMR spectra and (b) ^1^H MAS NMR spectrum of as-received sepiolite mineral.

The ^29^Si MAS, CP/MAS, and the ^1^H MAS NMR spectra of sepiolite in the as-received form are shown in Figure 3. In the ^29^Si MAS NMR spectrum three main resonances with similar intensities are observed to be located at −92.3 ppm, –94.8 ppm and −98.4 ppm. These three resonances can be assigned, on the basis of previous 2D ^1^H–^29^Si heteronuclear correlation (HETCOR) NMR studies,^37^ to near-edge (Type 2), center (Type 3), and edge (Type 1) Q^3^ Si sites of sepiolite, respectively (Figure 3a). The intensities and positions of these resonances are in accordance with the previous reports.^37,38^ A weak resonance positioned at −85.6 ppm is also detected, corresponding to about 5% of the total intensity. This resonance was assigned in previous studies to Q^2^ (Si–OH) Si sites^37,39^ and/or Q^3^ (Si-1Al) Si sites^40^ that are next nearest neighbors of Al atoms. However, the intensity of this resonance located at −85.6 ppm in the current system seems to be unchanged in the ^29^Si–^1^H CP/MAS spectrum (Figure 3a), which implies that this feature must be related to the Q^3^ (Si-1Al) Si sites rather than Q^2^ (Si–OH) Si sites. The ^1^H MAS NMR spectrum of the sepiolite consists primarily of two resonances located at 4.4 and 0.2 ppm (Figure 3b). The broad resonance at 4.4 ppm has been associated in the literature with the protons in zeolitic and coordinated water in the sepiolite structure while the sharp resonance at 0.2 ppm is attributed to the protons of the Mg–OH sites.^37,38^

**Figure 4:**
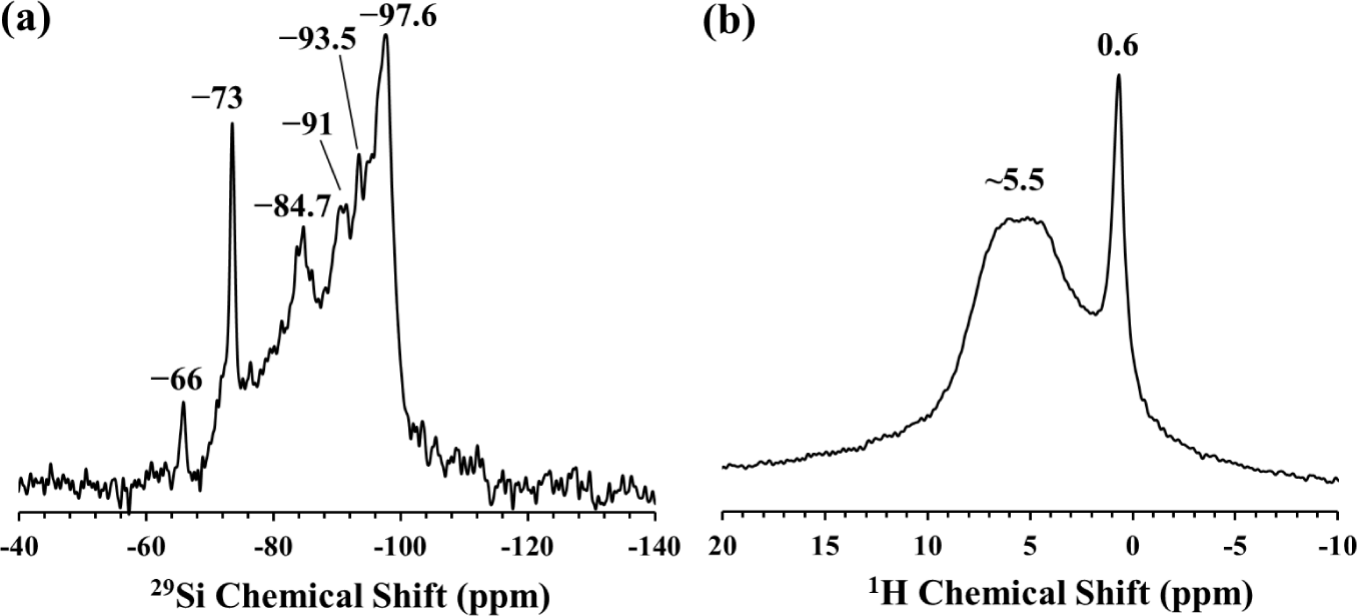
(a) ^29^Si MAS NMR spectrum and (b) ^1^H MAS NMR spectrum of sepiolite calcined at 750 °C for 1 h.

Calcination of the precursors is an important step in the synthesis of alkali-activated materials as it is known to enhance the solubility of the material in the alkali activator solution due to the partial amorphization of its structure.^41,42^ The structure of the as-received sepiolite is indeed observed to drastically change upon calcination at 750 °C. The ^29^Si and the ^1^H MAS NMR spectra of the calcined sepiolite are shown in Figure 4. The intense sharp resonances observed in the ^29^Si MAS for sepiolite (Figure 3a) lose intensity upon calcination, more profoundly for the near-edge and center Q^3^ Si sites, with all resonances shifting slightly to higher ppm values (Figure 4a). Overall, the ^29^Si MAS NMR spectrum displays a somewhat broadened form for the calcined sepiolite, mainly due to partial amorphization of the crystalline structure by the dehydroxylation process. Another significant effect of calcination is the appearance of new ^29^Si resonances centered at −66 and −73 ppm, indicating the formation of Q^0^ and Q^1^ Si units, possibly related to highly depolymerized calcium magnesium silicate or calcium silicate phases.^43^ The feature at −66 ppm seems to be related with monticellite phase detected in the corresponding XRD pattern in Figure 1b. However, a crystal phase matching with the resonance at −73 ppm (Q^1^ sites) could not be detected by XRD for calcined sepiolite, possibly due to its existence at very low concentrations or fine dispersion within the amorphous matrix.

These depolymerized units are expected to effectively interact with the sodium silicate solution, resulting in a geopolymer-like framework. On the other hand, the broad ^1^H resonance of sepiolite (Figure 3b) at 4.4 ppm significantly diminishes due to dehydroxylation upon calcination at 750 °C and a much broader resonance appears at around 5.5 ppm with significantly lower intensity (Figure 4b). The intensity of the sharp feature at 0.2 ppm for sepiolite also notably decreases in intensity upon calcination and shifts slightly downfield to 0.6 ppm. The ratio of the areas under the two resonances indicate that the relative concentration of Mg–OH sites increases over that of the structural water as a result of the thermal treatment.

### Structural Characterization of SAAM

**Figure 5:**
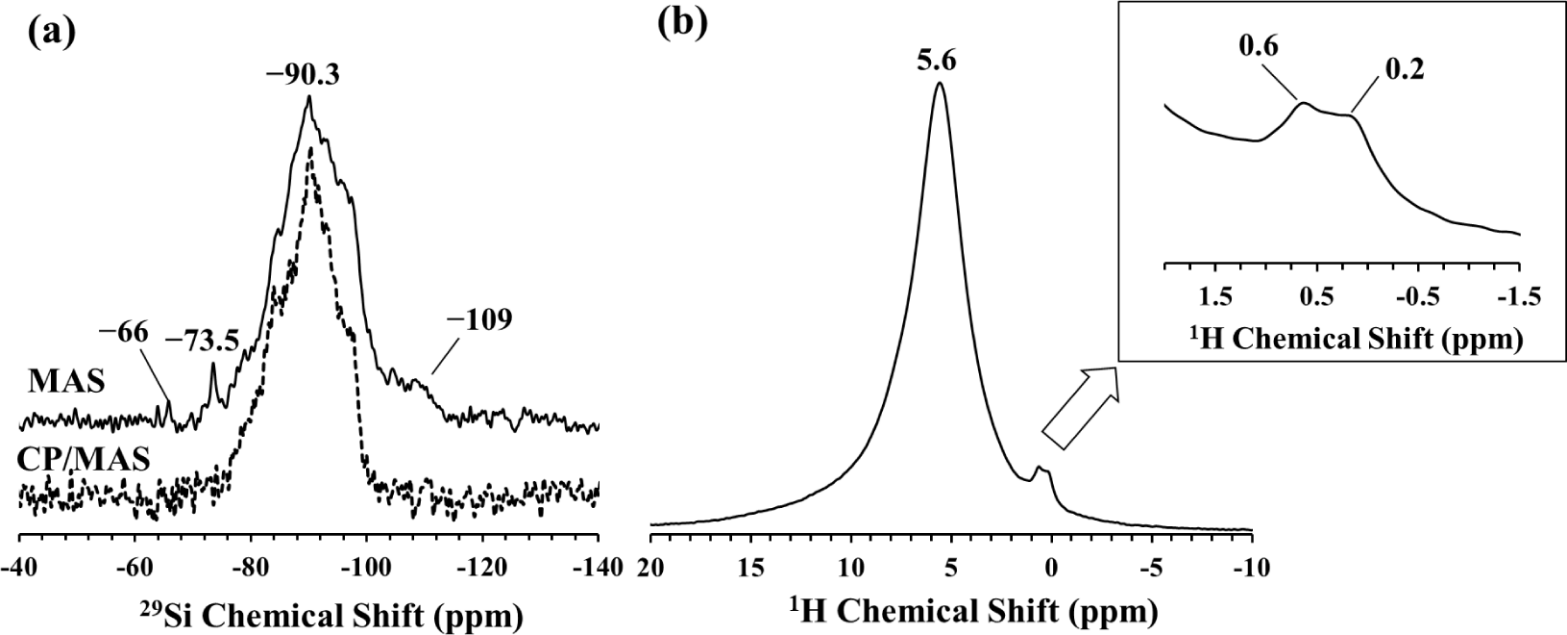
(a) ^29^Si MAS (solid line) and CP/MAS (dashed line) NMR spectra of SAAM. (b) ^1^H MAS NMR spectrum of SAAM. Inset shows an enlarged section of the upfield region of ^1^H MAS NMR spectrum.

The reaction of the calcined sepiolite with the sodium silicate solution (alkali-activation process) results in the transformation of the structure into a highly amorphized Na–Ca–Mg silicate product as a result of dissolution and reorganization processes in the alkaline environment. The ^29^Si and ^1^H MAS NMR and the ^29^Si CP/MAS spectra of this sepiolite based alkali-activated material (SAAM) are shown in Figure 5. The ^29^Si MAS NMR spectrum consists mainly of a broad resonance at −90.3 ppm, in strong resemblance with a previous report on a sepiolite-based geopolymeric material.^28^ Considering the ^29^Si chemical shifts in magnesium silicate systems, this broad resonance could be attributed primarily to Q^3^ (1Mg) Si sites.^44^ Broad and relatively weak shoulders in the ^29^Si MAS NMR spectrum near −80 ppm and −100 ppm can be assigned to Q^2^ (2Mg) and Q^3^ (1H) (hydrated silica), respectively. In traditional aluminosilicate geopolymer systems, the product of the geopolymerization reaction is a highly polymerized network formed by corner-shared AlO_4_ and SiO_4_ units displaying broad ^29^Si MAS NMR resonances positioned at around −90 ppm that indicate highly Al-substituted units such as Q^4^(2Al) or Q^4^(3Al) units.^26^ These highly polymerized structures exhibit strong mechanical properties that enable their use in various structural applications. Magnesium silicate analogs of geopolymers have also recently shown to be mechanically strong materials that are promising for MB elimination from wastewaters.^29,30^ Additionally, upon alkali activation of calcined sepiolite, a new broad resonance appears at −109 ppm in the ^29^Si MAS spectrum corresponding to the formation of fully polymerized Q^4^ Si species (Figure 5a) in SAAM. The ^29^Si CP/MAS spectrum lacks this feature indicating the absence of Si–H interactions in the highly polymerized SiO_2_-like regions within the magnesium silicate network. The ^29^Si MAS NMR spectrum of SAAM also exhibits sharp but rather weak resonances positioned at −66 and −73.5 ppm indicating the presence of small amounts of Q^0^ and Q^1^ Si species (Figure 5a), possibly originating as in the case of calcined sepiolite (Figure 4a), from unreacted calcium magnesium silicate crystals.^43^ However, the intensities of these resonances seem to be largely reduced in the SAAM compared to the ^29^Si MAS NMR spectrum of calcined sepiolite suggesting that consumption of these depolymerized units to form a more connected network in the former.

A previous study utilized ^25^Mg MAS NMR spectroscopy of a sepiolite based geopolymeric material to conclude that high alkaline conditions resulted in partial conversion of octahedrally cooordinated Mg in the starting material into Mg sites in tetrahedral coordination, signaling that the role of Mg could be similar to Al in the traditional aluminosilicate systems.^28^ In this scenario, the formation of Si–O–Mg linkages connecting MgO_4_ and SiO_4_ tetrahedral units, somewhat similar to T–O–T (T: Si or Al) sites in an aluminosilicate geopolymer, would be expected. It should also be noted that recent ultrahigh-field (35.2 T) ^25^Mg MAS NMR and triple-quantum MAS NMR spectroscopic results for Na_2_MgSi_3_O_8_, and K_2_MgSi_5_O_12_ glasses that are similar to SAAM system in terms of chemical composition demonstrated the coexistence of 4-fold and 5/6-fold- coordinated Mg sites.^45^

The ^1^H MAS NMR spectrum of SAAM is dominated by an intense resonance located at 5.6 ppm, corresponding to the structural water (Figure 5b). In analogous metakaolin-based aluminosilicate geopolymer systems, structural water is reported to be present within the cages of the framework together with alkali cations.^27^ Therefore, it would be expected to see a somewhat similar structural picture in the case of magnesium silicate SAAM system investigated here, as well. The proton environments for structural water in the SAAM appear to be less shielded compared to those of the as-received sepiolite where the ^1^H MAS NMR spectrum exhibited a resonance at 4.4 ppm (Figure 3b). This higher chemical shift of ^1^H could indicate formation of a network with larger pores in the geopolymer-like SAAM system as opposed to the size of channels in as-received sepiolite. The increase in the BET pore size as a result of alkali activation has in fact been experimentally reported for sepiolite based systems supporting this argument.^29^ The attribution of an increase in the ^1^H chemical shift of the structural water in SAAM with an enlargement in the pore size is also consistent with a previous report on water adsorption on graphene-based materials where the ^1^H NMR resonance was observed to move to lower chemical shift values as the pore size in the system was reduced.^46^ On the other hand, the relative concentration of the protons in Mg–OH environments in SAAM, characterized by resonances near 0.6 and 0.2 ppm (see inset of Figure 5b) appear to decrease markedly upon alkali activation compared to that in the as-received and calcined sepiolite samples (Figures 3b,4b).

### Structural Characterization of gCN-SAAM

**Figure 6:**
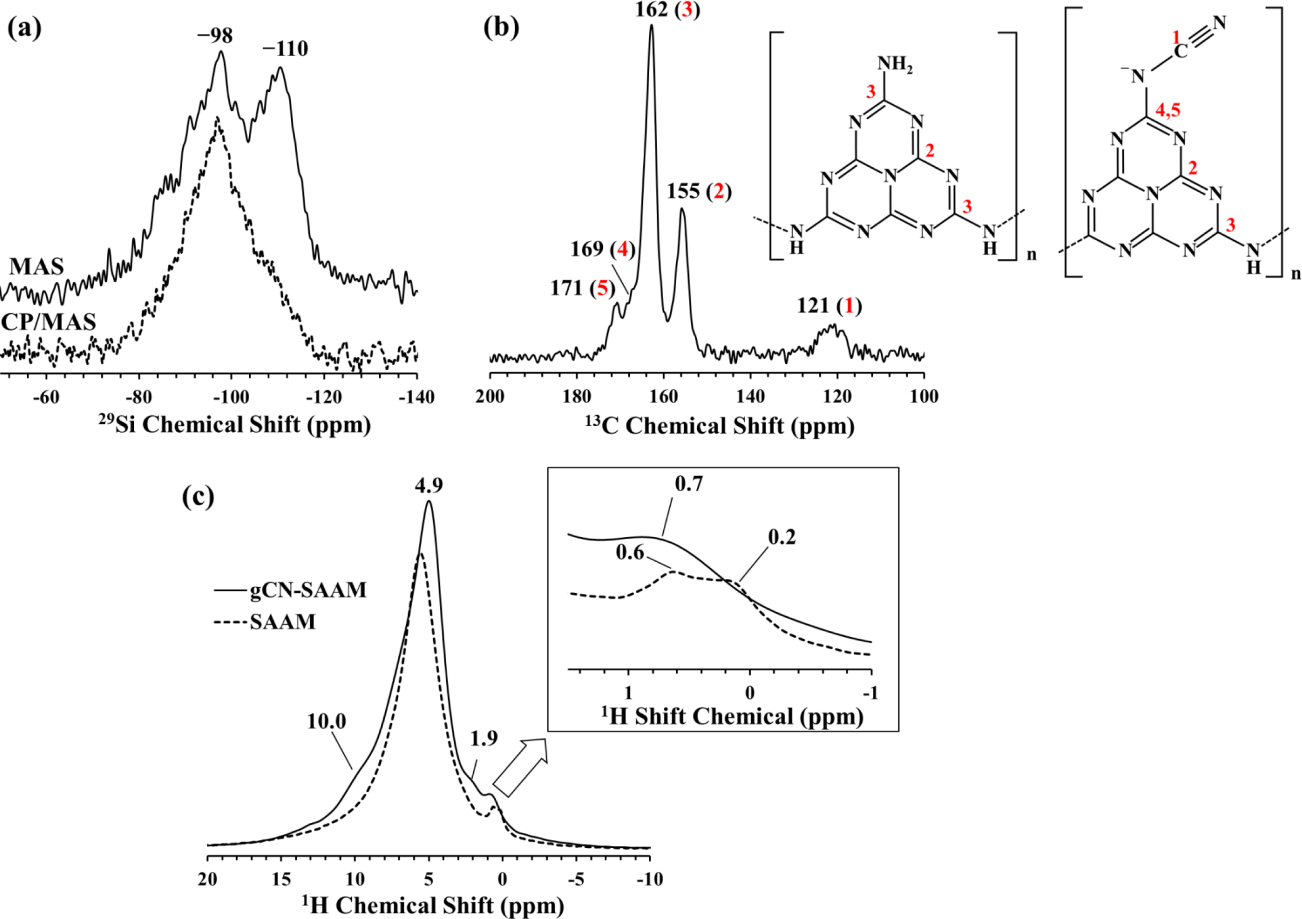
(a) ^29^Si MAS (solid line) and CP/MAS (dashed line) NMR spectra of gCN-SAAM. (b) ^13^C CP/MAS NMR spectrum of gCN-SAAM. (c) ^1^H MAS NMR spectrum of gCN-SAAM (solid line) in comparison with that of SAAM (dashed line). Inset shows an enlarged section of the upfield region of ^1^H MAS NMR spectra.

Treatment of SAAM with urea at 550 °C results in a hybrid g-C_3_N_4_-modified material, gCN-SAAM, where the structural prints of both SAAM and urea-based g-C_3_N_4_ are observed. The ^29^Si MAS and CP/MAS, ^13^C CP/MAS, and ^1^H MAS NMR spectra of gCN-SAAM are shown in Figure 6. Upon interaction with urea, the ^29^Si MAS NMR spectrum of SAAM displaying a broad resonance at −90.3 ppm (Figure 5a) transforms into one with two well resolved resonances, located at −98 ppm and −110 ppm in the ^29^Si MAS NMR spectrum for gCN-SAAM (Figure 6a) indicating the formation of a highly polymerized structure with enhanced connectivity in the latter. The resonance at −98 ppm corresponds to Q^3^ Si sites in gCN-SAAM while the strong resonance positioned at −110 ppm indicates a relative increase in the concentration of fully polymerized Q^4^ Si sites compared to that in the SAAM. Again, similar to SAAM, the intensity of the resonance at −110 ppm weakens in the ^29^Si CP/MAS NMR spectrum of gCN-SAAM, implying the absence of Si–H interactions in the highly polymerized SiO_2_-like regions within the magnesium silicate network.

The ^13^C CP/MAS NMR spectrum of gCN-SAAM (Figure 6b) displays two strong resonances at 162 and 155 ppm that can be readily attributed on the basis of previous studies to the C_2N-NHx_ and C_3N_ sites in the heptazine core structure of g-C_3_N_4_, confirming the formation and deposition of g-C_3_N_4_ species onto SAAM.^32,33,35^ Additionally, weak resonances are observed at 171, 169, and 121 ppm, corresponding to =N–C–^–^N, −N=C–^–^N, and –C≡N sites, respectively.^32^ The formation of −C≡N sites has been previously reported in polymeric carbon nitride systems modified with heteroatom dopants where NaOH assisted condensation process was reported to facilitate the formation of new functional groups, for instance deprotonating the terminal C–NH_2_ units to form −C≡N units.^35^ Considering the fact that SAAM has high alkali content, during its interaction with urea at 550 °C, it would be expected to have a similar alkali-assisted condensation process leading to these new functional groups.

The ^1^H MAS NMR spectrum of gCN-SAAM mainly exhibits an intense resonance at 4.9 ppm with minor resonances appearing located at ∼10.0 and 1.9 ppm (Figure 6c). To be able to better differentiate the structural changes that took place after urea treatment, the ^1^H MAS NMR spectrum of SAAM is shown together with gCN-SAAM in Figure 6c. The ^1^H MAS NMR spectrum of SAAM is normalized assuming that the broad resonance at 5.6 ppm corresponding to structural water would be mostly intact in the newly formed gCN-SAAM. Urea treatment seems to result in new resonances at 4.9 and 10.0 ppm in the ^1^H MAS NMR spectrum of gCN-SAAM. These resonances have been previously assigned to residual water and amino groups, respectively.^35^ A new resonance at 1.9 ppm also appears which can be possibly attributed to (isolated) silanols.^47,48^

### MB Adsorption Capacities of SAAM and gCN-SAAM

The MB uptake values of various adsorbents including g-C_3_N_4_ and its derivatives, aluminosilicate-based (geopolymers) and magnesium silicate-based alkali activated materials, sepiolite, and materials synthesized in this work (SAAM and gCN-SAAM) are shown together in Table 2 along with the corresponding experimental conditions. MB uptake depends on a variety of factors including type of raw material, chemical modifications, temperature, pH, surface area, initial MB concentration, and adsorbent dosage.^49^ All the adsorption experiments tabulated in Table 2 are carried out near ambient temperatures. It is seen that MB adsorption capacities of SAAM and gCN-SAAM are 105 and 420 mg L^–1^, respectively. The g-C_3_N_4_ modification increases the adsorption capacity of SAAM 4-fold, surpassing the performance of all of the adsorbents that are considered in this category. From this point forward, the discussion focuses on the structural changes in the local environments of the spent adsorbents to analyze the nature of the interactions and sites responsible for adsorption process.

**Table 2:** Comparison of the MB Adsoption Capacities of the Various Similar Adsorbents and the Materials Synthesized in This Work^a^

**Adsorbent type**	**q**_**e**_(mg g^–1^)	**Conditions**	**Source**
Bulk g-C_3_N_4_	1.28	C_0_ = 15 mg L^–1^ m_ads_ = 1 pH = N/A	(50)
C_3_N_4_ hollow tubes	7.22	C_0_ = 15 mg L^–1^ m_ads_ = 1 pH = N/A	(50)
Bulk g-C_3_N_4_	47.9	C_0_ = 70 mg L^–1^ m_ads_ = 0.2 pH = 7	(22)
C-doped g-C_3_N_4_	57.87	C_0_ = 15 mg L^–1^ m_ads_ = N/A pH = N/A	(51)
P-doped g-C_3_N_4_	100	C_0_ = 18 mg L^–1^ m_ads_ = N/A pH = 8	(52)
Hydoxyethylcellulose/silica/g-C_3_N_4_	132.45	C_0_ = 10 mg L^–1^ m_ads_ = 1.67 pH = N/A	(53)
Red-mud/metakaolin based geopolymer	2.27	C_0_ = 16 mg L^–1^ m_ads_ = 0.3 pH = N/A	(21)
g-C_3_N_4_/red-mud/metakaolin based geopolymer	170.9	C_0_ = 70 mg L^–1^ m_ads_ = 0.2 pH = 7	(22)
Bauxite residue-based geopolymer monolith	17.3	C_0_ = 75 mg L^–1^ m_ads_ = 7.18 pH = N/A	(20)
Sepiolite	79.37	C_0_ = 260 mg L^–1^ m_ads_ = N/A pH = 8.3	(54)
Sonicated sepiolite	128	C_0_ = 260 mg L^–1^ m_ads_ = N/A pH = 10	(54)
Alkali-activated sepiolite	74.64	C_0_ = 20 mg L^–1^ m_ads_ = 0.2 pH = 7	(29)
**SAAM (Sepiolite-based alkali activated Na–Ca–magnesium silicate)**	**105**	**C**_**0**_**= 80** mg L^–1^**m**_**ads**_**= 0.1 pH** = 7	**This study**
**gCN-SAAM (g-C**_**3**_**N**_**4**_**modified sepiolite-based alkali activated Na–Ca–magnesium silicate)**	**420**	**C**_**0**_**= 80** mg L^–1^**m**_**ads**_**= 0.1 pH** = 7	**This study**

a m_ads_ denotes the adsorbent dosage in g adsorbent/L MB solution.

### Structural Characterization of MB Adsorbed SAAM

**Figure 7:**
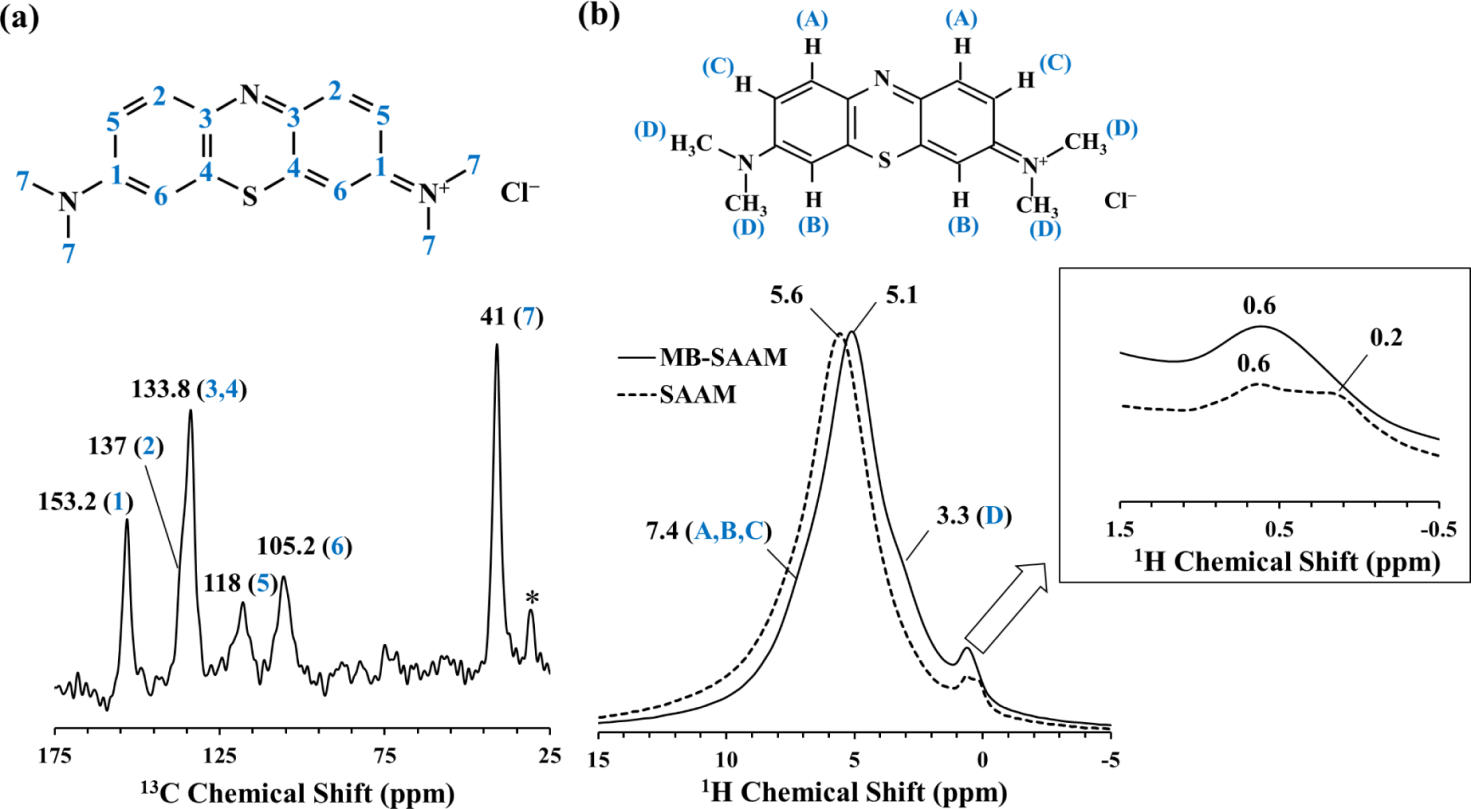
(a) ^13^C CP/MAS NMR spectrum of MB-SAAM. (b) ^1^H MAS NMR spectrum of MB-SAAM (solid lines) in comparison with that of SAAM (dashed lines). Inset shows an enlarged section of the upfield region of ^1^H MAS NMR spectra.

The ^13^C CP/MAS and the ^1^H MAS NMR spectra of the MB adsorbed SAAM (MB-SAAM) are shown in Figure 7. MB is the only source of ^13^C in this sample and the resonances observed at 153.2, 137, 133.8, 118, 105.2, and 41 ppm are fingerprints of MB molecules^55^ adsorbed on SAAM (see Figure 7a for peak assignments).

The ^1^H MAS NMR spectrum of the MB-SAAM is compared in Figure 7b with that of SAAM to see the changes originating from MB adsorption. The main resonance at 5.6 ppm in SAAM shifts to 5.1 ppm in MB-SAAM indicating significant interaction of the water molecules within the geopolymer-like framework upon the adsorption of MB molecules. In contrast, the lower field Mg–OH resonance at 0.6 ppm seems to remain intact. Additionally, two new resonances appear in the ^1^H MAS NMR spectrum of the MB-SAAM at ∼7.4 and 3.3 ppm that can be assigned to H sites in MB molecules,^56^ thus demonstrating the adsorption of MB molecules on the surface of SAAM (Figure 7b).

**Figure 8:**
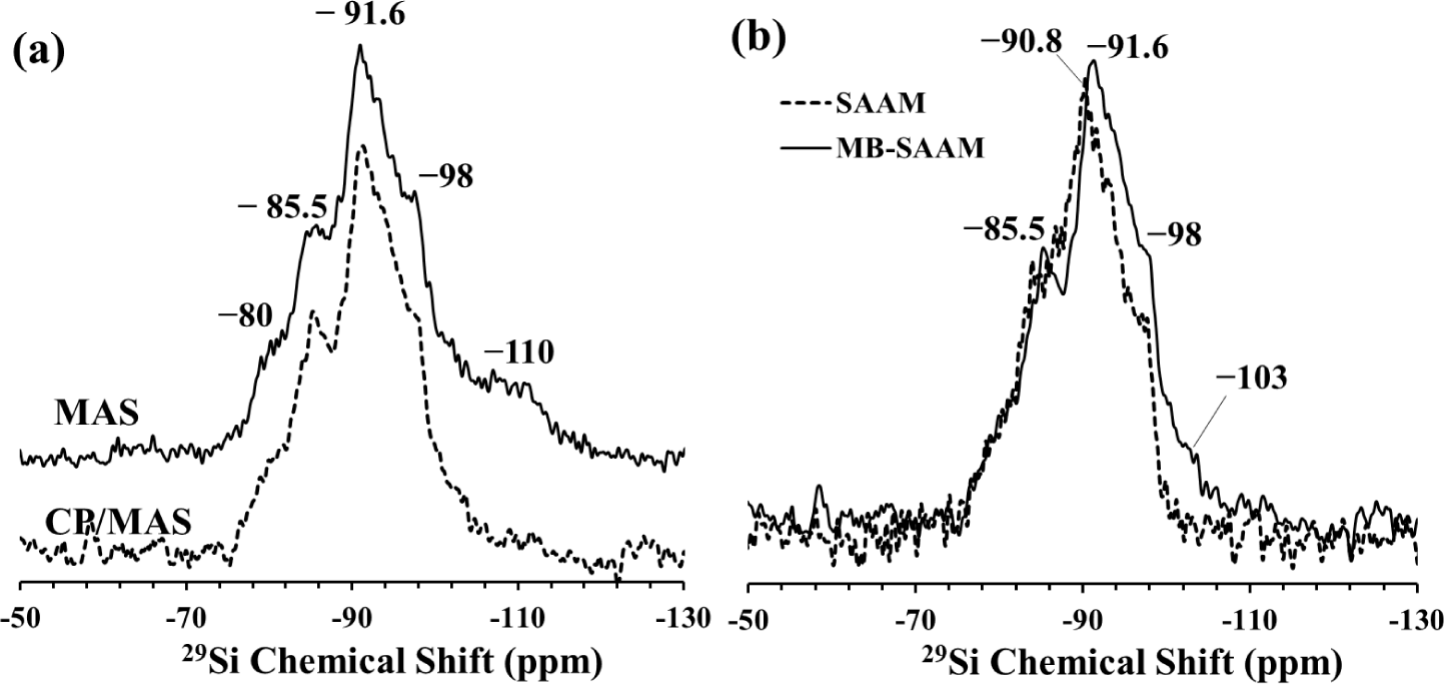
(a) ^29^Si MAS (solid line) and ^29^Si CP/MAS (dashed line) NMR spectra of MB-SAAM. (b) ^29^Si CP/MAS NMR spectrum of MB-SAAM (solid line) in comparison with that of SAAM (dashed lines).

The ^29^Si MAS NMR spectrum of MB-SAAM display an intense and broad resonance peaking at −91.6 ppm (Figure 8a). The ^29^Si CP/MAS NMR spectrum of MB-SAAM seems to be almost identical to its ^29^Si MAS NMR counterpart except for lacking the resonance at −110 ppm that is indicative of highly polymerized Q^4^ Si sites (Figure 8a). A comparison of the ^29^Si CP/MAS NMR spectrum of MB-SAAM with that of SAAM (Figure 8b) shows a clear shift of the center of gravity of the line shape to lower ppm values (higher field) with enhancement of intensity for resonances near −98 and −103 ppm upon interaction with MB suggesting clear correlation between the Si sites in SAAM and the protons of the MB molecule.

### Structural Characterization of MB Adsorbed gCN-SAAM

**Figure 9:**
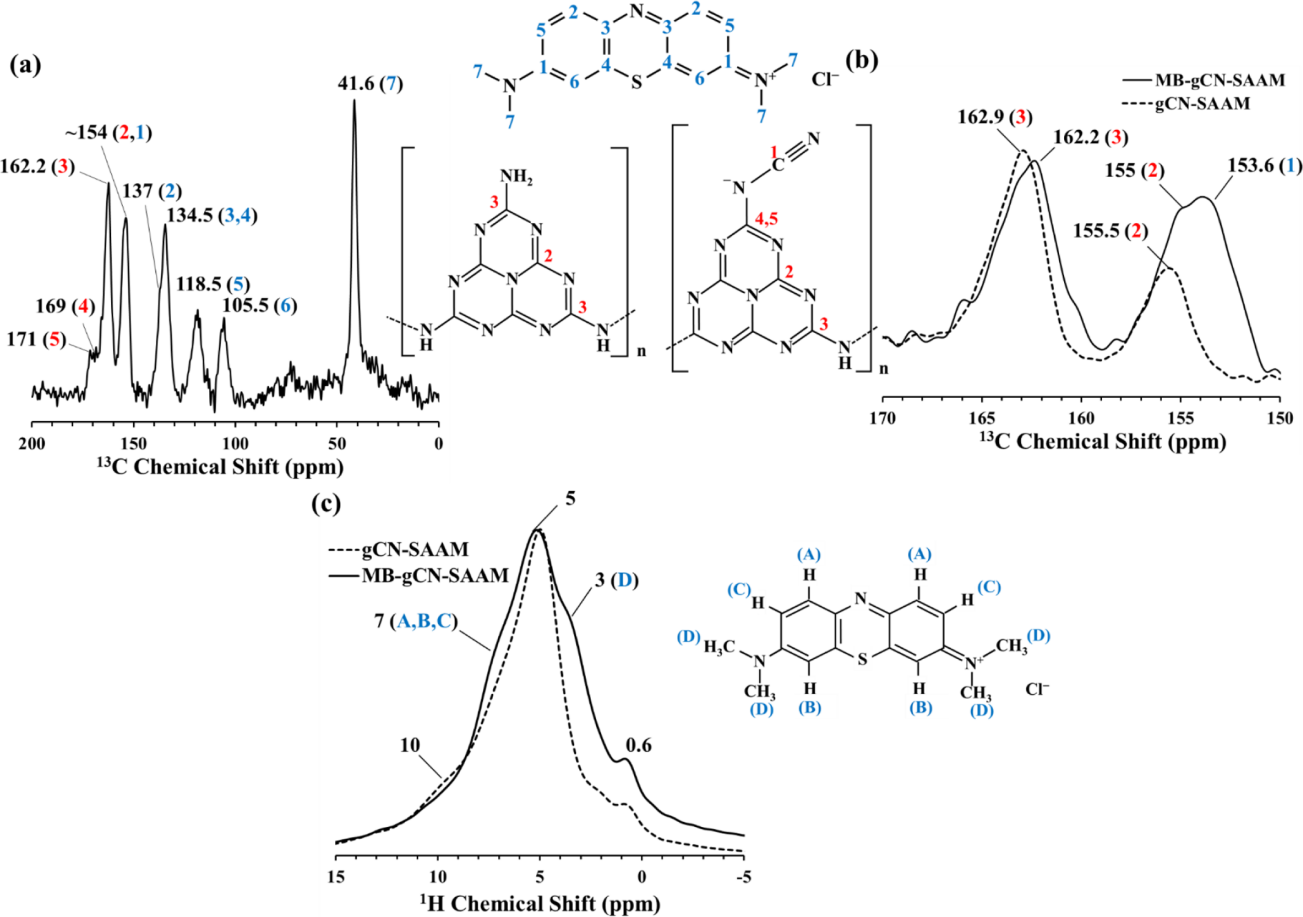
(a) ^13^C CP/MAS NMR spectrum of MB-gCN-SAAM. (b) Zoomed-in version of ^13^C CP/MAS NMR spectrum of MB-gCN-SAAM (solid line) together with that of gCN-SAAM (dashed line). (c) ^1^H MAS NMR spectrum of MB-gCN-SAAM (solid line) together with that of gCN-SAAM (dashed line).

The ^13^C CP/MAS and the ^1^H MAS NMR spectra of MB adsorbed gCN-SAAM (MB-gCN-SAAM) are shown in Figure 9. The structural fingerprints of different C sites of MB can be clearly seen in the ^13^C CP/MAS NMR spectrum displaying resonances in the region between 155 and 40 ppm, coexisting together with the resonances originating from C sites of g-C_3_N_4_ regions of gCN-SAAM (Figure 9a). Figure 9b shows a zoomed-in version of the downfield region of the ^13^C CP/MAS spectrum of the MB-gCN-SAAM together with that of gCN-SAAM. The resonances positioned at 162.2 and 155 ppm are indicative of C_2N-NHx_ and C_3N_ sites in the heptazine units of g-C_3_N_4_ regions of gCN-SAAM. The positions of these two resonances shift to lower ppm values compared to those of gCN-SAAM (162.9 and 155.5 ppm) and the intensity near 155 ppm increases significantly as a result of interaction with MB. These results indicate that the C2 and C3 sites of gCN-SAAM plays a role in adsorption process. On the other hand, the resonances at 171 and 169 ppm, corresponding to =N–C–^–^N, –N=C–^–^N sites, respectively, remain intact showing no significant sign of interaction.

The ^1^H MAS NMR spectrum of the MB-gCN-SAAM is compared in Figure 9c with that of gCN-SAAM. The strongest resonance in the proton spectrum of MB-gCN-SAAM located near 5 ppm is mainly correlated with the structural water present in the SAAM regions of the hybrid gCN-SAAM material. The fingerprints of the protons of the adsorbed MB molecule are also evident from the resonances appearing at 7 and 3 ppm, indicating CH and N(CH_3_) sites of MB, respectively.^56^ The interaction of this new adsorbent with MB seems to result in the disappearance of the resonance positioned at 10 ppm, which likely represents the amino groups of the g-C_3_N_4_ regions of gCN-SAAM. This finding suggests that the MB molecules interact with NH_2_ sites of gCN-SAAM. In contrast, the resonance at 0.6 ppm corresponding to the Mg–OH sites remain mainly intact, suggesting no clear sign of interaction of MB with these sites.

**Figure 10:**
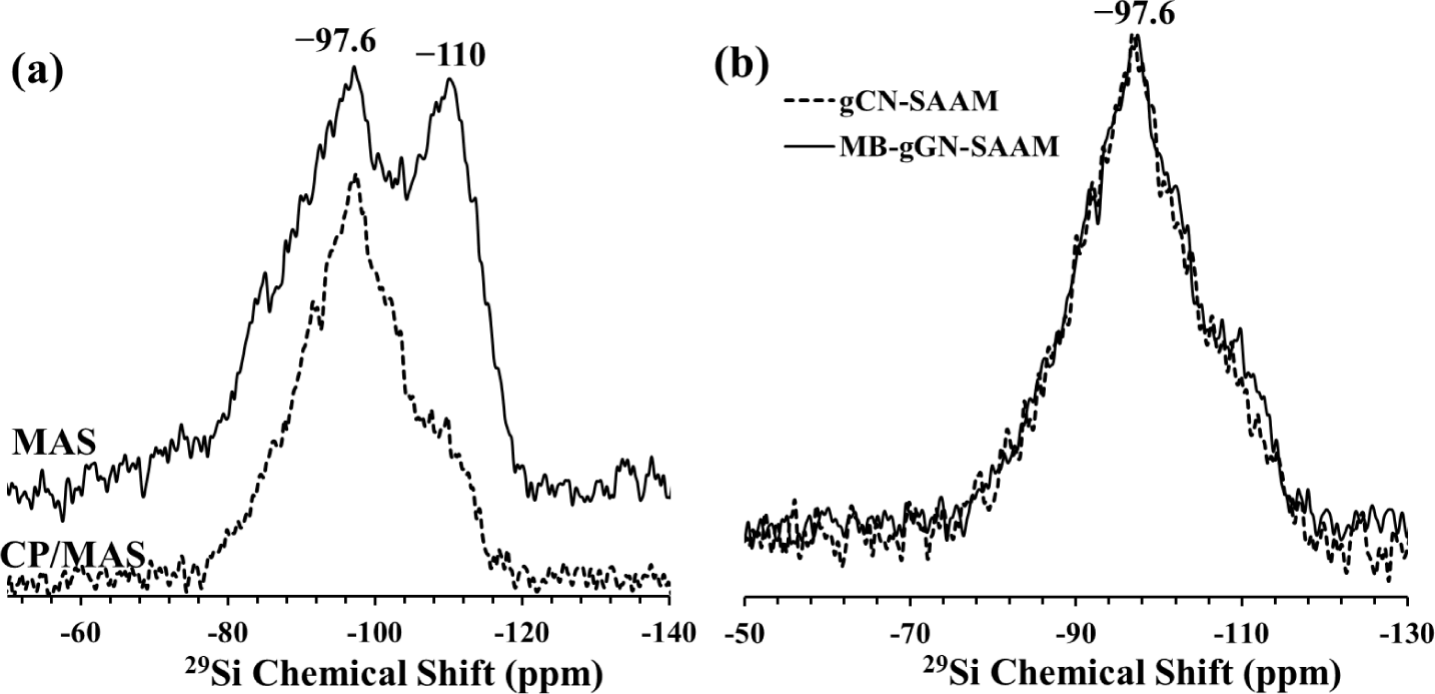
(a) ^29^Si MAS (solid line) and CP/MAS (dashed line) NMR spectra of MB-gCN-SAAM. (b) ^29^Si CP/MAS NMR spectra of MB-gCN-SAAM (solid line) in comparison with that of gCN-SAAM (dashed line).

The ^29^Si MAS and the ^29^Si CP/MAS NMR spectra of the MB-gCN-SAAM are shown in Figure 10a. These two spectra are almost identical with the corresponding spectra of the adsorbent before interaction with MB, namely gCN-SAAM (Figure 6a). To be able to more clearly demonstrate chemical environments of the Si sites with MB adsorption, ^29^Si CP/MAS NMR spectrum of the MB-gCN-SAAM sample is compared with the cross polarized spectrum of gCN-SAAM in Figure 10b. Again, and in contrast with the case of MB adsorption on SAAM, the ^29^Si CP/MAS spectra before and after MB adsorption on gCN-SAAM are practically identical, showing no evidence of interaction of Si sites of with MB molecules.

Physical properties of an adsorbent, such as surface area, pore volume, and pore size, along with surface chemistry and functional groups, are known to play important roles in efficient adsorption processes. Although good adsorption performance is usually correlated with a high specific surface area, gCN-SAAM, which exhibits superior adsorption performance in this study, has a BET surface area of only 28.2 m^2^/g.

The current study involves the adsorption of methylene blue, which exists in aqueous solution in its cationic form. Therefore, it is expected that a negatively charged adsorbent surface would attract cationic dye molecules. In a recent study involving the utilization of g-C_3_N_4_ for MB adsorption, electrostatic attraction between the negative surface charge of g-C_3_N_4_ and the positive charge of MB (N^+^ in the xanthene ring) was reported to occur at lower MB concentrations, while π–π interactions between the substrate and the surface were observed at higher MB concentrations.^57^ Another recent study involving g-C_3_N_4_-deposited red-mud metakaolin geopolymer as an adsorbent for MB removal similarly indicated that the negative surface charge of the adsorbent, together with a variety of functional groups, facilitated the attraction of MB molecules to the surface.^22^

In a separate work investigating the adsorption and photocatalytic properties of tris(4-aminophenyl) amine-based polyimide (TP)/g-C_3_N_4_ composites for the removal of rhodamine B, another cationic dye, it was reported that although pure g-C_3_N_4_ exhibited the highest surface area compared to pure TP and the TP/g-C_3_N_4_ composite, its adsorption performance was the lowest among the materials studied.^58^ These studies highlight the important role of surface charge and chemistry in dye adsorption processes.

## Conclusions

This study introduces a novel adsorbent (gCN-SAAM) synthesized based on a simple thermal process involving condensation of urea-derived g-C_3_N_4_ onto an amorphous magnesium silicate system (SAAM) synthesized by alkali activation of sepiolite. This procedure yields a hybrid adsorbent with exceptional MB adsorption capability, boosting a remarkable adsorption capacity of 420 mg g^–1^ under ambient conditions—a 4-fold increase compared to the unmodified magnesium silicate adsorbent. To elucidate the complex structures of these adsorbents and interaction of MB with these adsorbent systems, with a focus on local Si, H, and C environments, solid-state ^29^Si (MAS and CP/MAS), ^1^H MAS, and ^13^C CP/MAS NMR spectroscopy are conducted on both the pristine and MB-saturated adsorbents. The ^29^Si NMR results demonstrate that the silicate network of SAAM consists mainly of Q^3^ Si units with significant amount of structural water and Mg–OH sites. A clear modification of the ^29^Si CP MAS and the ^1^H MAS NMR spectrum is observed upon MB adsorption signaling the direct involvement of the Si sites and of the water molecules within the geopolymer-like framework of the SAAM in the adsorption process.

Treatment of SAAM with urea to form gCN-SAAM results in enhanced polymerization of Q^n^ species with a notable upfield shift in ^29^Si MAS NMR spectrum compared to SAAM, a strong enhancement in Q^4^ Si sites and the disappearance of some of the Mg–OH units suggesting interaction of these sites with g-C_3_N_4_. Condensation of g-C_3_N_4_ units onto gCN-SAAM is clearly observed based on the NMR signatures of heptazine core, namely C_2N-NHx_ and C_3N_ sites, as well as −C≡N and amino groups. Upon contact with MB, Si sites of gCN-SAAM remain completely intact signaling no interaction, while the NH_2_ groups disappear and the ^13^C chemical shifts corresponding to the C_2N-NHx_ and C_3N_ sites move to higher field, signifying the key role of g-C_3_N_4_ in the MB adsorption process of gCN-SAAM.

Utilizing multinuclear solid-state NMR spectroscopy to explore the interactions between complex–structured adsorbents and methylene blue (MB), as adopted in this work, is essential for gaining a mechanistic understanding of the adsorption process. However, this approach has not been commonly used in previous studies. Further research is needed to fully explore its potential. For instance, future studies could focus on two-dimensional (2D) heteronuclear correlation (HETCOR) NMR spectroscopy, which could provide valuable insights into the interactions and connectivity between MB and magnesium silicate adsorbents at the molecular level. Additionally, complementary Density Functional Theory (DFT) studies would be highly beneficial for modeling the surface properties of the adsorbents as well as their interactions with dye molecules.

The exceptional methylene blue (MB) adsorption capacity of gCN-SAAM suggests its strong potential for removing a wider range of pollutants from contaminated water such as dyes, heavy metals, and other industrial chemicals in wastewater. Given the global demand for more sustainable wastewater treatment technologies, the sustainable and cost-effective nature of the synthesis method adopted here presents an opportunity for the development of new materials to address dye pollution problem.

Although g-C_3_N_4_ and alkali-activated materials are each known for their thermal and chemical stability, future work should focus on conducting multiple adsorption and desorption cycles on these new hybrid systems. This would simulate real-world conditions where adsorbents are exposed to varying loading conditions over time. Additionally, detailed studies on adsorption kinetics and isotherms are crucial for optimizing the material’s performance and assessing its practical applicability in large-scale wastewater treatment systems.
